# Different Risk Factors for Very Low Birth Weight, Term-Small-for-Gestational-Age, or Preterm Birth in Japan

**DOI:** 10.3390/ijerph15020369

**Published:** 2018-02-21

**Authors:** Naomi Tamura, Tomoyuki Hanaoka, Kumiko Ito, Atsuko Araki, Chihiro Miyashita, Sachiko Ito, Hisanori Minakami, Kazutoshi Cho, Toshiaki Endo, Kazuo Sengoku, Katsuhiko Ogasawara, Reiko Kishi

**Affiliations:** 1 Center for Environmental and Health Sciences, Hokkaido University, Kita 12, Nishi 7, Kita-ku, Sapporo 060-0812, Japan; ntamura@cehs.hokudai.ac.jp (N.T.); tmykhanaoka@me.com (T.H); kumiko27@med.hokudai.ac.jp (K.I.); AAraki@cehs.hokudai.ac.jp (A.A.); miyasita@med.hokudai.ac.jp (C.M.); vzbghjn@den.hokudai.ac.jp (S.I.); oga@hs.hokudai.ac.jp (K.O.); 2 Graduate School of Health Sciences, Hokkaido University, Kita 8, Nishi 5, Kita-ku, Sapporo 060-0812, Japan; 3 Graduate School of Medicine, Hokkaido University, Kita 15, Nishi 7, Kita-ku, Sapporo 060-8638, Japan; 4 Department of Obstetrics and Gynecology, Graduate School of Medicine, Hokkaido University, Kita 15, Nishi 7, Kita-ku, Sapporo 060-8638, Japan; minasho@med.hokudai.ac.jp (H.M.); chotarou@med.hokudai.ac.jp (K.C.); 5 Department of Obstetrics and Gynecology, School of Medicine, Sapporo Medical University, Chuo-ku, Sapporo 060-8556, Japan; endot@sapmed.ac.jp; 6 Department of Obstetrics and Gynecology, Asahikawa Medical University, 2-1-1-1, Midorigaoka Higashi, Asahikawa 078-8510, Japan; ksen@asahikawa-med.ac.jp; 7 Faculties of Health Science, Hokkaido University, Kita12, Nishi5, Kita-ku, Sapporo 060-0812, Japan

**Keywords:** birth cohort, parental factor, fetal growth restriction, socioeconomic condition.

## Abstract

From 1985 to 2013, the mean birth weight of infants in Japan decreased from 3120 g to 3000 g, and the low-birth-weight rate among live births increased from 6.3% to 9.6%. No prospective study has elucidated the risk factors for poor fetal growth and preterm birth in recent Japanese parents, such as increased parental age, maternal body figure, assisted reproductive technology (ART), and socioeconomic status. Participants were mother–infant pairs (*n* = 18,059) enrolled in a prospective birth cohort in Hokkaido, Japan from 2002 to 2013. Parental characteristics were obtained via self-reported questionnaires during pregnancy. Medical records helped identify very-low-birth-weight (VLBW; <1500 g), term-small-for-gestational-age (term-SGA), and preterm-birth (PTB; <37 weeks) infants. We calculated relative risks (RRs) for PTB, VLBW, and term-SGA birth based on parental characteristics. The prevalence of PTB, VLBW, and term-SGA was 4.5%, 0.4%, and 6.5%, respectively. Aged parents and ART were risk factors for PTB and VLBW. Maternal alcohol drinking during pregnancy increased the risk; a parental educational level of ≥16 years reduced risk of term-SGA. Maternal pre-pregnancy BMI of <18.5 kg/m^2^ increased the risk of PTB and term-SGA. The RR for low BMI was highest among mothers who have low educational level. Among various factors, appropriate nutritional education to maintain normal BMI is important to prevent PTB and term-SGA in Japan.

## 1. Introduction

Poor fetal growth, such as low birth weight (LBW), small-for-gestational age (SGA), and preterm birth (PTB) have serious health effects not only during the neonatal period and infancy, but also later in life [1]. LBW, SGA, and PTB are suggested to be associated with neurological, metabolic, and cardiovascular morbidities. Despite being an advanced developed country, Japan had the second highest prevalence of low-birth-weight infants in 2016 among the Organisation for Economic Co-operation and Development (OECD) countries [2]. From 1985 to 2013, the mean birth weight of infants in Japan decreased from 3120 g to 3000 g, and the LBW rate among live births increased from 6.3% to 9.6% [3]. Over a similar period, the PTB rate increased from 4.5% in 1990 to 5.8% in 2013 [3].

Reported maternal risk factors for PTB and SGA include maternal young and advanced age, low maternal body mass index (BMI), short stature, low weight, mother born as SGA, and cigarette smoking [4,5,6,7]. Furthermore, previous studies conducted in Europe and the USA have suggested a significant association between infant birth weight and maternal socioeconomic status [8,9]. A previous descriptive study suggested that the increased prevalence was due to increasing multiple births and fertility treatments, increased maternal age, and an increased rate of smoking among young women in Japan [10]. Recently, a study reported that fetuses are at risk of LBW, because Japanese women desire a slim figure and limited weight gain during pregnancy [11]. In addition, a previous study conducted in Japan also reported that PTB and SGA were associated with parental socioeconomic status [12]. The OECD report noted that Japan’s relative poverty rate—the proportion of people with net income below a defined threshold—was 16.1% in 2012. Currently, it remains well above the OECD average [13]. Paternal influence as a cause of poor fetal growth and preterm births was also suspected [14]. In a systematic review, advanced paternal age and low educational level were associated with LBW and PTB [15,16,17]. However, no study has been conducted to consider the association between parental risk factors, such as increased parental age, maternal body figure, family socioeconomic status, and their life style, and LBW, SGA, and PTB among a prospective birth cohort study in Japan.

In this study, we aimed to determine parental characteristics, such as increased parental age, maternal body figure, family socioeconomic status, their life style, and medical treatment as risk factors for VLBW, term-SGA, and PTB in Japan.

## 2. Materials and Methods 

### 2.1. Participants

**Figure 1:**
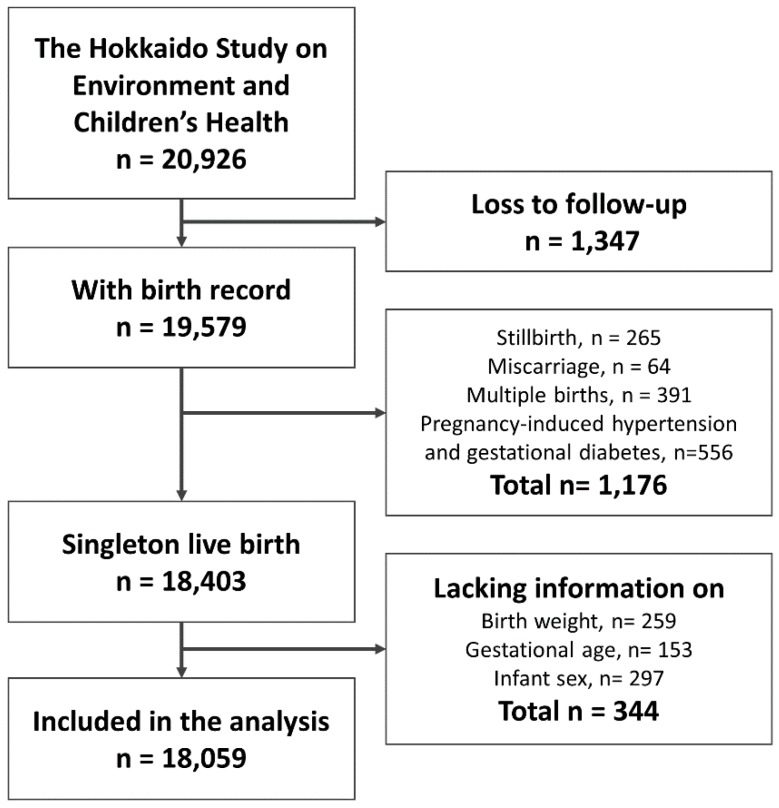
Flow chart of participants included in the statistical analysis.

The Hokkaido Study on Environment and Children’s Health is an ongoing cohort study that began in 2002. The study’s aims and methods were described in three previous profile papers and are only briefly discussed in this study [18,19,20]. From February 2003 to March 2012, the Hokkaido (large-scale) cohort enrolled women during early pregnancy (13 weeks of gestational age) who visited the maternity unit in one of the 37 associated hospitals and clinics in the Hokkaido Prefecture for prenatal health. The 37 associated hospitals and clinics cover the whole Hokkaido area. The cohort consists of 20,926 pregnant women. Among them, 1347 were lost to follow-up before giving birth (Figure 1). As this study focused on the outcomes of VLBW, term-SGA, and PTB, we excluded women who had miscarriages, stillbirths, multiple births, pregnancy-induced hypertension, and gestational diabetes (*n* = 1176). Thus, we eliminated the pathological causes of VLBW, term-SGA, and PTB, which could have masked and underestimated the risk factors of parental characteristics. Participants lacking information on the three outcomes of interest were also excluded (*n* = 344). Thus, a total of 18,059 participants were included in the statistical analysis that assessed the associations between parental factors and VLBW, term-SGA, and PTB.

### 2.2. Baseline Questionnaire

At study entry, the participants completed a self-administered questionnaire covering information on parental characteristics, including maternal and paternal age at entry (≤24, 25–34, ≥35 years), maternal BMI before pregnancy (<18.5, 18.5–24.9, 25–29.9, ≥30 kg/m^2^), maternal and paternal previous medical history, regular use of any supplement(s), maternal active smoking during the 1st trimester, maternal passive smoking during the 1st trimester, paternal smoking habit until the 1st trimester, parental drinking habit until the 1st trimester, and use of any assisted reproductive technologies (ART). We used paternal and maternal education level (≤9, 10–12, 13–15, ≥16 years of education), and family household income (<3.0, 3.0–4.9, 5.0–7.9, ≥8 million yen), as socioeconomic indicators because these are important in young adulthood [21,22].

### 2.3. Outcomes of Poor Fetal Growth and Preterm Birt

Information on sex of infant, gestational age (days), and birth weight (g) were obtained at delivery from the medical records. We adopted three outcomes for poor fetal growth and preterm birth: VLBW, term-SGA, and PTB to evaluate “birth weight” and “preterm birth” as separate variables [23]. PTB was defined as live birth at <37 completed gestational weeks; VLBW was defined as a birth weight <1500 g; and term-SGA was defined as a birth weight lower than the 10th percentile of the normative reference birth weight, according to gestational age, sex, and parity, in infants live born at >37 gestational weeks. To calculate term-SGA, we used the database for birth weight published by the Japan Pediatric Society as a reference [24], because Asian people are smaller than Caucasian people.

### 2.4. Data Analysis

**Figure 2:**
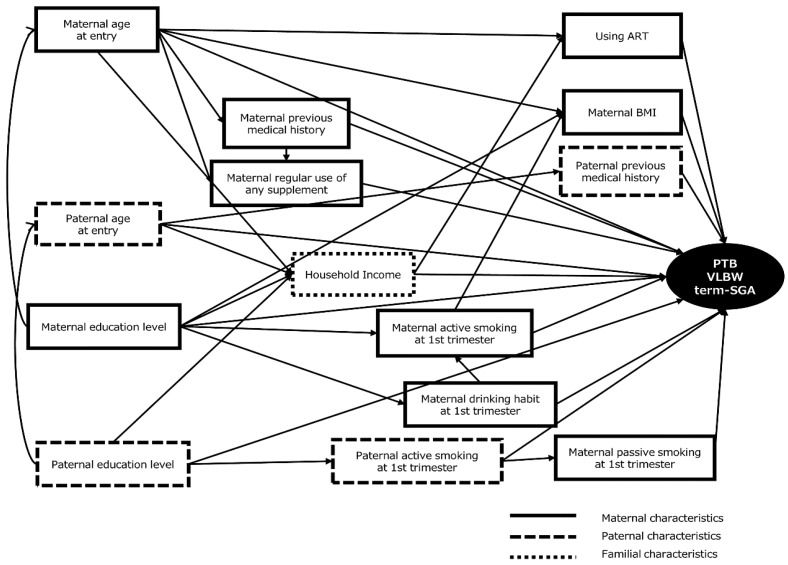
DAG for PTB, VLBW, and term-SGA. ART, assisted reproductive technology; BMI, body mass index; PTB, preterm birth; term-SGA, term- small for gestational age; VLBW, very low birth weight.

Continuous data are presented as the mean and standard deviation (SD). Categorical data are presented as frequency and percentage. The Chi-square test was used to assess associations between VLBW, term-SGA, and PTB, and parental factors. The relative risks (RR) of VLBW, term-SGA, and PTB according to parental characteristics were estimated using multiple Generalized Linear Models (distribution: binominal, link function: logarithm). The models with each factor were adjusted according to maternal age and educational level. A directed acyclic graph (DAG) was constructed to identify a minimum set of confounding adjustment (Figure 2) [25,26]. We selected the set of covariates for each factor that were regarded as the main exposure to effect on RRs of outcome such as VLBW, term-SGA, and PTB. The confounding factor(s) 1 (F1) that directly connected to both outcome and main exposure was (were) included. In addition, the confounding factor(s) 2 (F2) that directly connected to both outcome and F1 was (were) included. The mediating factor(s) that was (were) between outcome and main exposure was (were) excluded. The collider(s) that was (were) affected by both main exposure and F1 or F2 or outcome was (were) excluded. This shows the hypothesis of relationships between maternal and paternal, and socio-economic characteristics and outcome. We selected the set of covariates for each factor that was regarded as the main exposure that affected RRs of VLBW, term-SGA, and PTB. We excluded mediators and colliders from the covariates. We examined two-way interactions between each parental risk factor as a main exposure and covariations. When *P*_interaction_ was less than 0.05, then each covariate was stratified for groups and a risk factor analysis of the main exposure was conducted. When we calculated RRs, we used the majority group (for parental age, maternal BMI, parity, maternal regular use of any supplement, parental education level, parental occupation, and household income) and the lowest risk group (for maternal active and passive smoking, paternal smoking habit, parental drinking habit, parental previous medical history, and maternal regular use of any medicine) as the reference categories. To estimate the RRs of term-SGA, only participants who delivered term infants were included in the analysis. 

Since there were many missing values for parental factors, we imputed missing values using partial least squares regression. Two-sided values of *p* < 0.05 were considered statistically significant. All statistical estimates were calculated using JMP Clinical 5 statistical software (SAS Institute Inc., Cary, NC, USA).

### 2.5. Ethical Approval

All participating mothers provided written informed consent before participation in the Hokkaido Study. The study protocol was approved by the ethics review board for epidemiological studies at Hokkaido University Graduate School of Medicine (March 31, 2003) and the Hokkaido University Center for Environmental and Health Sciences (reference no.14, March 22, 2012), in accordance of with principles of the Declaration of Helsinki.

## 3. Results

Maternal and paternal characteristics are shown in Table 1. The mean gestational age was 39.2 (SD: 1.5) weeks and the mean birth weight was 3039.0 (SD: 411.2) g. PTB was observed in 805 (4.5%) of the 18,059 births, VLBW in 74 (0.4%), and term-SGA in 1192 (6.6%). Male infants comprised 50.4% (*n* = 9096) of the total sample. There were 6621 (36.7%) primipara mothers. 

Table 2 presents the association between VLBW and parental characteristics. In the adjusted model based on DAG, the RR of VLBW was significantly higher among infants with older mothers (maternal age ≥35 vs. 25–34 years: RR = 1.90; 95% CI, 1.10–3.29) and among infants with older fathers (paternal age ≥35 vs. 25–34 years: RR = 2.02; 95% CI, 1.22–3.35). Maternal BMI (>30 kg/m^2^ vs. 18.5–25) and used ART were significantly associated in the crude model and adjusted models, but they were not statistically significant in the adjusted model.

Table 3 presents the RRs of term-SGA and parental characteristics. In the adjusted model of term-SGA, compared to the infants whose mothers were in the standard BMI (18.5–25 kg/m^2^) group, the RR was significantly higher for infants whose mothers were in the lowest BMI (<18.5 kg/m^2^) category (RR = 1.77; 95% CI, 1.55–2.03) and was significantly lower for infants whose mothers were in the BMI (25.0–29.9 kg/m^2^) category (RR = 0.70, 95% CI, 0.53–0.93). The RR of being born term-SGA was significantly higher in infants whose mothers continued to drink during the first trimester (RR = 1.57; 95% CI, 1.33–1.85) compared with those whose mothers never drank alcohol, and whose mothers regularly used any supplement (RR = 1.16; 95% CI, 1.03–1.30). The RR of being born term-SGA was significantly lower in infants whose mothers had >16 years (vs. 10–12 years) of education (RR = 0.76; 95% CI, 0.61–0.94). The RR of being born term-SGA was significantly lower in infants whose fathers had >16 years (vs. 10–12 years) of education (RR = 0.86; 95% CI, 0.75–1.00). Maternal passive smoking was not significantly associated after adjustment. 

Table 4 presents the prevalence and RR of PTB with parental characteristics. In the adjusted model based on DAG, the RR of PTB was significantly higher among infants with older mothers (maternal age ≥ 35 vs. 25–34 years: RR = 1.45; 95% confidence interval (CI), 1.23–1.71); among infants whose mothers had a low (<18.5 kg/m^2^) BMI compared with a standard (18.5–25 kg/m^2^) BMI (RR = 1.45; 95% CI, 1.21–1.73); infants whose mothers had any previous medical history (RR = 1.17; 95% CI, 1.02–1.35); and for those whose mothers used ART (RR = 1.76; 95% CI, 1.36–2.29). The RR of PTB was also significantly higher for infants whose father’s highest level of education was 13–15 years compared with 10–12 years (RR = 1.26; 95% CI, 1.06–1.50). On the other hand, the RR of PTB was significantly lower for infants whose parent’s household income was less than 3 million yen compared with 3–5 million yen (RR = 0.80; 95% CI, 0.64–1.00). Paternal age, paternal smoking habit, paternal previous medical history, and maternal educational level were not significantly associated after adjustment.

We found significant interaction effects between pre-pregnancy BMI and maternal educational level (*P_interaction_* = 0.01) using ART and maternal age at entry (*P_interaction_* = 0.02) for PTB, and using maternal educational level and maternal age at entry (*P_interaction_* = 0.03) for VLBW. Table 5 presents the result of stratified analysis by interaction covariates to examine parental risk factors. After stratification, RRs of maternal educational levels were not significant for stratified analysis of maternal age at entry for VLBW. Similarly, the RR of low (<18.5 kg/m^2^ vs. 18.5–25 kg/m^2^) to PTB was significantly associated among mothers’ educational level ≤9 years, 9–12, and >16 years, and among them, RR was the highest in the group of ≤9 years (RR = 2.31; 95% CI, 1.15–4.65). After stratification by maternal age at entry, the RR of PTB was significantly associated only among infants whose mothers used ART (RR = 2.06; 95% CI, 1.45–2.93) in the 25–35 years old mothers group. 

Supplemental tables S1 to S3 present the results of the analysis, including substituted values of parental characteristics. The distribution of the parental characteristics in Supplemental tables S1 and S2 are comparable to those presented in Table 1, Table 2, Table 3 and Table 4, respectively. As shown in Supplemental table S3, paternal smoking increased the RR of PTB, whereas lower household income (<3 million yen vs. 3–5 million yen) reduced the RR significantly. For VLBW and term-SGA, the results were comparable between models with and without imputed values.

## 4. Discussion

The mean birth weight and gestational age in this study were comparable to the data obtained from recent vital statistics of Japan [3]. We evaluated the non-pathological maternal and paternal factors with three proxy indicators for poor fetal growth and preterm birth: VLBW, term-SGA, and PTB. The results showed that various parental factors were associated with each of these three outcomes and suggested that the life style and socioeconomic conditions in young Japanese women affected VLBW, term-SGA, and PTB in different ways. In short, higher maternal and paternal age and using ART were the main risk factors for VLBW and PTB, whereas life styles such as maternal alcohol drinking habits during the 1st trimester increased, but maternal and paternal educational level of ≥16 years decreased the risk for term-SGA. In addition, maternal pre-pregnancy BMI of <18.5 kg/m^2^ was a risk factor for both term-SGA and PTB. Maternal and paternal factors were significantly correlated with each other, so that minimum and exact covariate factors should be selected for the adjustment model [14]. Thus, DAG model was used to determine the effects of parental factors on VLBW, term-SGA, and PTB in this study.

Higher maternal and paternal age and using ART were the main risk factors for VLBW and PTB. Only 0.4% of infants were born with VLBW. Although the sample size was small, maternal and paternal ages of >35 years were significantly associated with VLBW. Advanced maternal age (≥35 years) has been previously reported as a significant risk factor for VLBW [27]. In this study, advanced paternal age (≥35 years vs. 25–34 years) was associated with PTB and VLBW. Advanced paternal age has been reported as a risk factor for PTB—which is related to VLBW—even if maternal age is <35 years [15]. Further studies that measure paternal involvement are needed to better assess the role of fathers in enhancing prenatal health behaviors and pregnancy outcomes. Because of the lifestyle of modern Japanese people, birth to advanced aged parents and the accompanying use of ART will continue to increase. For aged parents and when using ART, advanced knowledge on PTB and VLBW are needed, even if no visible pathological cause was observed.

In this study, the higher BMI, the higher the RR of VLBW (>30 kg/m^2^ vs. 18.5–25 kg/m^2^) in the crude and adjusted models, although the negative effect was insignificant in the DAG model. Studies conducted in the US and European countries have reported that a high pre-pregnancy BMI has a disadvantageous effect on fetal growth [28,29]. The US and European countries considered a BMI of >30 kg/m^2^ as the standard criterion for high BMI. Previous studies reported that Asians have a lower BMI, but a higher percentage of body fat than Caucasians [30,31]. However, only 2.0% of 18,059 mothers had a BMI of >30 kg/m^2^ in the present study, and the proportion of VLBW was only 0.4%. Hence, we were unable to detect the negative effect of >30 kg/m^2^ BMI. Notably, Table 2 and Table 4 present that low maternal BMI before pregnancy significantly increased the risk of PTB and term-SGA. Moreover, the results of interaction effects between pre-pregnancy BMI and maternal educational level for PTB presents that the RR of low BMI (<18.5 kg/m^2^ vs. 18.5–25 kg/m^2^) was highest in the group with educational level of ≤9 years (RR = 2.31; 95% CI, 1.15–4.65). Han et al. reported in a meta-analysis that a low BMI in pregnant women significantly increased the risk of VLBW, PTB, and intrauterine growth restriction [32]. Moreover, nutritional deficiency during pregnancy should be considered among Japanese women. The Ministry of Health, Labour, and Welfare recommends that pregnant women consume 1800–2200 kcal/day. However, in 2011, the National Health and Nutrition Survey showed that the average intake among pregnant women was only 1665 kcal/day [33]. Adequate knowledge on taking essential nutrition during pre-pregnancy and pregnancy should be provided.

Previous studies suggested that smoking during pregnancy decreased newborn birth weight and gestational age [34]. However, in this study, active smoking was insignificantly associated with VLBW, term-SGA, and PTB, which was examined during early pregnancy (13 weeks of gestational age), so that quitting smoking during pregnancy reduced the risk of VLBW, term-SGA, and PTB. The effect of maternal smoking during the 1st trimester is unclear and has not been extensively studied [35,36,37]. If mothers continue to actively smoke until the third trimester, then the negative impact on newborn birth weight and gestational age is inevitable. Indeed, we have reported that birth weight reduction showed a dose-dependent decreasing relationship with maternal prenatal cotinine levels during the third trimester in the same cohort [38].

In this study, maternal and paternal education (≥16 years vs. 10–12 years) significantly reduced the RR of term-SGA. As mentioned, not only maternal but also paternal educational level could be an important factor in avoiding the risks associated with term-SGA. A previous study in Japan reported that parental educational level was significantly associated with SGA [12]. Education represents knowledge-related assets and indicates both economic resources and status. Socioeconomic factors may affect term-SGA via smoking and alcohol consumption [21], and indeed, alcohol drinking habit increased the risk of SGA in this study. Moreover, maternal education was significantly associated with smoking and alcohol consumption during pregnancy (data not shown). A smaller proportion of mothers in the highest educational categories were active smokers or alcohol drinkers during pregnancy (*p* < 0.01, data not shown). Education could be an important factor to avoid the risk factors associated with VLBW, term-SGA, and PTB. 

We excluded women who had stillbirths, multiple births, pregnancy-induced hypertension, and gestational diabetes. Most stillbirth and multiple-birth infants show VLBW, term-SGA, or preterm characteristics. Pregnancy-induced hypertension and gestational diabetes have already been reported to have a decreased or increased effect on gestational age and birth weight [39]. Furthermore, maternal chronic hypertension and pregnancy-induced hypertension have been associated with pre-pregnancy diabetes mellitus and gestational diabetes, respectively [40,41]. These pathological factors associated with VLBW, term-SGA, and PTB could mask and underestimate the parental characteristics. Thus, in this study, we excluded mothers with hypertension and gestational diabetes, so that we could determine the impact of parental characteristics as a risk factor for VLBW, term-SGA, and PTB even without pathological basis.

The strengths of this study are as follows: first, it was a prospective birth cohort study design. Participants were recruited in a general hospital setting, such as local obstetric clinics. Second, the loss-to-follow-up rate was only 5.9%. Third, we initiated a DAG model to identify a minimum set of confounding adjustment, to avoid over-adjustment of our multiple analysis model. Limitations of this study included the following: first, the amount of missing data was relatively large. For example, 17.9% of household income data were missing. However, to estimate the effects of missing values, we imputed values using partial least square regression. The distributions of parental characteristics were comparable, and RRs were not different between raw and imputed data. Second, the participants of this cohort study were pregnant women who had visited hospitals or clinics within the Hokkaido Prefecture only. However, the participating hospitals and clinics were local medical institutions and distributed throughout the prefecture, accounting for approximately 40% of the institutes with delivery units in this prefecture [20]. Moreover, the distribution of participant characteristics was close to that of the overall Japanese population [42,43], suggesting the results are generalizable. Third, possible residual confounding factors may also exist. Besides parental factors obtained using the questionnaire, residual confounding factors may have had an effect on VLBW, term-SGA, and PTB.

## 5. Conclusions

This study showed that different parental factors were associated with three proxy indicators of poor fetal growth and preterm birth: VLBW, term-SGA, and PTB in Japan. These results suggest that both maternal and paternal advanced age and using ART are predictors of VLBW and PTB. Maternal alcohol drinking habit increased the risk of term-SGA, whereas both maternal and paternal high educational levels were protective to term-SGA infants. In addition, maternal pre-pregnancy BMI of <18.5 kg/m^2^ was a risk factor for both term-SGA and PTB. Moreover, the results of interaction effects between pre-pregnancy BMI and maternal educational level for PTB presents that the RR of low BMI was highest in the group with educational level of ≤9 years.

## Figures and Tables

**Table 1:** Parental characteristics of participants.

	*N* (%)			*N* (%)	
**Maternal Characteristics**			**Paternal Characteristics**		
**Age at entry (years old)**			**Age at entry (years old)**		
≤24	2634	(14.6)	≤24	1313	(7.3)
25–34	12,284	(68.0)	25–34	10,393	(57.6)
≥35	3132	(17.3)	≥35	5340	(29.6)
Missing	9	(0.0)	Missing	1013	(5.6)
**Prepregnancy BMI ^1^ (kg/m^2^)**			**Smoking habit during 1st trimester**		
<18.5	2996	(16.6)	No	5021	(27.8)
18.5–24.9	12,309	(68.2)	Yes	10,071	(55.8)
25.0–29.9	1342	(7.4)	Missing	2967	(16.4)
≥30.0	362	(2.0)	**Previous medical history**		
Missing	1050	(5.8)	No	11,348	(62.8)
**Active smoking during 1st trimester**			Yes	6705	(37.1)
No	14,425	(79.9)	Missing	6	(0.0)
Yes	1975	(10.9)	**Paternal educational level (years)**		
Missing	1659	(9.2)	≤9	1329	(7.4)
**Passive smoking during 1st trimester**			10–12	7149	(39.6)
No	4857	(26.9)	13–15	4011	(22.2)
Yes	11,327	(62.7)	≥16	4518	(25.0)
Missing	1875	(10.4)	Missing	1052	(5.8)
**Drinking habit during 1st trimester**			**Familial characteristics**		
Never	6688	(37.0)	**Household income (million yen)**		
Ex-drinker	8446	(46.8)	<3.0	3391	(18.8)
Current drinker	2009	(11.1)	3.0–4.9	6605	(36.6)
Missing	916	(5.1)	5.0–7.9	3736	(20.7)
**Previous medical history**			≥8	1086	(6.0)
No	9780	(54.2)	Missing	3241	(17.9)
Yes	8260	(45.7)			
Missing	19	(0.1)			
**Regular use of any supplement**					
No	12,549	(69.5)			
Yes	5492	(30.4)			
Missing	18	(0.1)			
**Using ART** ^2^					
No	16,565	(91.7)			
Yes	712	(3.9)			
Missing	782	(4.3)			
**Maternal educational level (years)**					
≤9	934	(5.2)			
10–12	7512	(41.6)			
13–15	6968	(38.6)			
≥16	1852	(10.3)			
Missing	793	(4.4)			

^1^ ART: assisted reproductive technology; ^2^ BMI: body mass index.

**Table 2:** The prevalence and the relative risk of very low birth weight (*n* = 74) stratified by parental characteristics ^5^.

		VLBW		Non VLBW			Crude ^2^				Adjustment Model ^2,3^				Based on DAG Model ^2,4^			
								95%CI				95%CI				95%CI		
		*N*	%	*N*	%	*p*-Value ^1^	RRs	Lower	Upper	*p*-Value	RRs	Lower	Upper	*p*-Value	RRs	Lower	Upper	*p*-Value
**Maternal characteristics**																		
**Age at entry (years old)**																		
	<24	9	0.3	2625	99.7	**0.03**	1.00	0.49	2.05	1.00	1.05	0.50	2.22	0.90	1.05	0.50	2.22	0.90
	25–34	42	0.3	12,242	99.7		Reference				Reference				Reference			
	≥35	21	0.7	3111	99.3		**1.96**	**1.16**	**3.31**	**0.02**	**1.90**	**1.10**	**3.30**	**0.03**	**1.90**	**1.10**	**3.29**	**0.03**
**Prepregnancy BMI (kg/m^2^)**																		
	<18.5	11	0.4	2980	99.6	0.14	1.01	0.52	1.94	0.99	1.05	0.54	2.03	0.89	1.13	0.58	2.21	0.72
	18.5–24.9	45	0.4	12,255	99.6		Reference				Reference				Reference			
	25.0–29.9	7	0.5	1334	99.5		1.43	0.64	3.16	0.40	1.39	0.62	3.08	0.44	1.51	0.68	3.36	0.34
	≥30.0	4	1.1	358	98.9		**3.02**	**1.09**	**8.35**	**0.07**	**2.85**	**1.02**	**7.93**	**0.08**	2.46	0.76	7.92	0.18
**Active smoking during 1st trimester**																		
	No	55	0.4	14,370	99.6	0.41	Reference				Reference				Reference			
	Yes	10	0.5	1965	99.5		1.33	0.68	2.60	0.42	1.41	0.71	2.76	0.34	1.41	0.72	2.77	0.34
**Passive smoking during 1st trimester**																		
	No	14	0.3	4843	99.7	0.18	Reference				Reference				Reference			
	Yes	49	0.4	11,278	99.6		1.50	0.83	2.72	0.16	1.84	0.97	3.47	0.05	1.40	0.44	4.43	0.57
**Drinking habit during 1st trimester**																		
	Never	19	0.3	6669	99.7	0.17	Reference				Reference				Reference			
	Ex-drinker	39	0.5	8407	99.5		1.63	0.94	2.81	0.07	1.60	0.92	2.78	0.09	1.58	0.91	2.73	0.10
	Current drinker	10	0.5	1999	99.5		1.75	0.82	3.76	0.16	1.70	0.79	3.67	0.19	1.73	0.81	3.72	0.17
**Previous medical history**																		
	No	38	0.4	9742	99.6	0.81	Reference				Reference				Reference			
	Yes	34	0.4	8226	99.6		1.06	0.67	1.68	0.81	0.81	0.35	1.88	0.61	1.04	0.64	1.69	0.87
**Regular use of any supplement**																		
	No	50	0.4	12,499	99.6	0.98	Reference				Reference				Reference			
	Yes	22	0.4	5470	99.6		1.01	0.61	1.66	0.98	0.95	0.56	1.61	0.84	**1.16**	**1.03**	**1.30**	**0.02**
**Using ART**																		
	No	61	0.4	16,504	99.6	**0.01**	Reference				Reference				Reference			
	Yes	7	1.0	705	99.0		**2.67**	**1.23**	**5.82**	**0.03**	**2.36**	**1.06**	**5.25**	**0.06**	2.17	0.92	5.13	0.11
**Maternal educational level (years)**																		
	≤ 9	4	0.4	930	99.6	0.62	1.07	0.38	3.04	0.90	1.12	0.39	3.22	0.84	1.07	0.38	3.04	0.90
	10–12	30	0.4	7482	99.6		Reference				Reference				Reference			
	13–15	23	0.3	6945	99.7		0.83	0.48	1.42	0.49	0.81	0.47	1.40	0.44	0.83	0.48	1.42	0.49
	≥16	10	0.5	1842	99.5		1.35	0.66	2.76	0.42	1.29	0.62	2.65	0.50	1.35	0.66	2.76	0.42
**Paternal characteristics**																		
**Age at entry (years old)**																		
	<24	5	0.4	1308	99.6	**0.02**	1.18	0.38	3.64	0.77	1.28	0.49	3.33	0.63	1.18	0.38	3.64	0.77
	25–34	30	0.3	10,363	99.7		Reference				Reference				Reference			
	≥35	31	0.6	5309	99.4		**2.01**	**1.22**	**3.32**	**<0.01**	1.73	0.97	3.09	0.07	**2.02**	**1.22**	**3.35**	**<0.01**
**Smoking habit during 1st trimester**																		
	No	16	0.3	5005	99.7	0.21	Reference				Reference				Reference			
	Yes	46	0.5	10,025	99.5		1.43	0.81	2.53	0.20	1.60	0.89	2.88	0.11	1.45	0.80	2.62	0.20
**Previous medical history**																		
	No	46	0.4	11,302	99.6	0.86	Reference				Reference				Reference			
	Yes	26	0.4	6679	99.6		0.96	0.59	1.55	0.86	0.90	0.54	1.49	0.67	0.98	0.59	1.63	0.93
**Paternal educational level (years)**																		
	≤9	6	0.5	1323	99.5	0.98	1.15	0.48	2.78	0.76	1.18	0.48	2.89	0.73	1.15	0.48	2.78	0.76
	10–12	28	0.4	7121	99.6		Reference				Reference				Reference			
	13–15	15	0.4	3996	99.6		0.95	0.51	1.79	0.88	0.98	0.52	1.86	0.96	0.95	0.51	1.79	0.88
	≥16	18	0.4	4500	99.6		1.02	0.56	1.84	0.95	0.88	0.46	1.70	0.70	1.02	0.56	1.84	0.95
**Familial characteristics**																		
**Household income (million yen)**																		
	<3.0	8	0.2	3383	99.8	0.32	0.58	0.26	1.27	0.15	0.58	0.26	1.29	0.16	0.57	0.25	1.29	0.16
	3.0–4.9	27	0.4	6579	99.6		Reference				Reference				Reference			
	5.0–7.9	19	0.5	3717	99.5		1.24	0.69	2.23	0.47	1.18	0.64	2.15	0.60	1.14	0.62	2.10	0.68
	≥8	4	0.4	1082	99.6		0.90	0.32	2.57	0.84	0.81	0.28	2.38	0.69	0.76	0.26	2.28	0.62

^1^: Calculated by Chi-square test. ^2^: Calculated by generalized liner regression models. ^3^: Adjustment model was adjusted by maternal age, and maternal education. ^4^: Based on DAG model was as Figure 2 as follows: Maternal age was adjusted by maternal educational level; Maternal BMI was adjusted by maternal age, maternal active smoking, and maternal educational level; Maternal active smoking at 1st trimester was adjusted by maternal educational level, and maternal drinking habit during 1st trimester; Maternal passive smoking at 1st trimester was adjusted by paternal active smoking during 1st trimester and parental educational level; Maternal drinking habit at 1st trimester was adjusted by maternal educational level; Maternal previous medical history was adjusted by maternal age, and maternal educational level; Maternal regular use of any supplement was adjusted by maternal age, maternal previous medical history, and maternal educational level; Using ART was adjusted by maternal age, maternal educational level, and household income. Maternal educational level was not adjusted by anything. Paternal age was adjusted by paternal educational level. Paternal active smoking at 1st trimester was adjusted by maternal educational level. Paternal previous medical history was adjusted by paternal age and paternal educational level. Paternal educational level was not adjusted by anything. Household Income was adjusted by parental age and parental educational level. ^5^: Term- small for gestational age (SGA) case group was compared with a control group of infants born at 37–41 weeks’ gestational age. ART: assisted reproductive technology; BMI: body mass index; CI: Confidence Interval; DAG: directed acyclic graph; RRs: Relative Risks; VLBW: Very Low Birth Weight.

**Table 3:** The prevalence and the relative risk of term-small for gestational age (*n* = 1192) stratified by parental characteristics ^5^.

		Term-SGA		Non Term-SGA			Crude ^2^				Adjustment Model ^2,3^				Based on DAG Model ^2,4^			
								95%CI				95%CI				95%CI		
		N	%	N	%	*p*-Value ^1^	RRs	Lower	Upper	*p*-Value	RRs	Lower	Upper	*p*-Value	RRs	Lower	Upper	*p*-Value
**Maternal characteristics**																		
**Age at entry (years old)**																		
	<24	158	6.2	2375	93.8	0.34	0.89	0.75	1.05	0.15	0.85	0.72	1.02	0.07	0.85	0.72	1.02	0.07
	25–34	825	7.0	10,919	93.0		Reference				Reference				Reference			
	≥35	208	7.1	2726	92.9		1.01	0.87	1.17	0.90	0.99	0.85	1.15	0.86	0.99	0.85	1.15	0.86
**Prepregnancy BMI (kg/m^2^)**																		
	<18.5	305	10.8	2522	89.2	**<0.01**	**1.75**	**1.54**	**1.99**	**<0.01**	**1.79**	**1.58**	**2.04**	**<0.01**	**1.77**	**1.55**	**2.03**	**<0.01**
	18.5–24.9	726	6.2	11,056	93.8		Reference				Reference				Reference			
	25.0–29.9	55	4.3	1220	95.7		**0.70**	**0.54**	**0.92**	**<0.01**	**0.69**	**0.53**	**0.90**	**<0.01**	**0.70**	**0.53**	**0.93**	**<0.01**
	≥30.0	16	4.6	329	95.4		0.75	0.46	1.22	0.23	0.75	0.46	1.22	0.22	0.76	0.46	1.25	0.26
**Active smoking during 1st trimester**																		
	No	958	7.0	12,801	93.0	0.95	Reference				Reference				Reference			
	Yes	130	6.9	1748	93.1		0.99	0.83	1.19	0.95	0.98	0.81	1.17	0.79	0.96	0.80	1.16	0.70
**Passive smoking during 1st trimester**																		
	No	289	6.2	4358	93.8	**0.03**	Reference				Reference				Reference			
	Yes	775	7.2	10,026	92.8		**1.15**	**1.01**	**1.31**	**0.03**	1.12	0.98	1.29	0.09	1.18	0.92	1.52	0.20
**Drinking habit during 1st trimester**																		
	Never	392	6.2	5970	93.8	**<0.01**	Reference				Reference				Reference			
	Ex-drinker	536	6.6	7538	93.4		1.08	0.95	1.22	0.25	1.09	0.96	1.24	0.16	1.09	0.96	1.23	0.20
	Current drinker	186	9.7	1734	90.3		**1.57**	**1.33**	**1.86**	**<0.01**	**1.56**	**1.32**	**1.85**	**<0.01**	**1.57**	**1.33**	**1.85**	**<0.01**
**Previous medical history**																		
	No	642	6.9	8721	93.1	0.71	Reference				Reference				Reference			
	Yes	549	7.0	7290	93.0		1.02	0.92	1.14	0.71	1.00	0.89	1.12	0.94	0.99	0.88	1.11	0.87
**Regular use of any supplement**																		
	No	201	1.8	11,164	98.2	0.07	Reference				Reference				Reference			
	Yes	390	7.4	4848	92.6		1.11	0.99	1.25	0.08	1.12	0.99	1.26	0.08	**1.16**	**1.03**	**1.30**	**0.02**
**Using ART**																		
	No	1081	6.8	14,751	93.2	0.42	Reference				Reference				Reference			
	Yes	50	7.6	604	92.4		1.12	0.85	1.47	0.43	1.10	0.83	1.44	0.52	1.07	0.79	1.44	0.68
**Maternal educational level (years)**																		
	≤9	74	8.3	813	91.7	**<0.01**	1.23	0.98	1.56	0.09	1.27	1.00	1.61	0.06	1.23	0.98	1.56	0.09
	10–12	486	6.8	6704	93.2		Reference				Reference				Reference			
	13–15	475	7.2	6154	92.8		1.06	0.94	1.20	0.35	1.04	0.92	1.18	0.53	1.06	0.94	1.20	0.35
	≥16	91	5.1	1681	94.9		**0.76**	**0.61**	**0.94**	**0.01**	**0.73**	**0.59**	**0.91**	**<0.01**	**0.76**	**0.61**	**0.94**	**0.01**
**Paternal characteristics**																		
**Age at entry (years old)**																		
	<24	90	7.1	1173	92.9	0.82	1.05	0.85	1.30	0.64	1.19	0.92	1.54	0.18	1.03	0.83	1.28	0.77
	25-34	673	6.8	9265	93.2		Reference				Reference				Reference			
	≥35	354	7.0	4708	93.0		1.03	0.91	1.17	0.61	1.01	0.87	1.16	0.93	1.04	0.92	1.18	0.54
**Smoking habit during 1st trimester**																		
	No	309	6.4	4500	93.6	0.09	Reference				Reference				Reference			
	Yes	688	7.2	8901	92.8		1.12	0.98	1.27	0.09	1.09	0.96	1.25	0.19	1.07	0.94	1.23	0.32
**Previous medical history**																		
	No	736	6.8	10,113	93.2	0.34	Reference				Reference				Reference			
	Yes	456	7.2	5909	92.8		1.06	0.94	1.18	0.34	1.02	0.91	1.15	0.71	1.03	0.91	1.16	0.65
**Paternal educational level (years)**																		
	≤ 9	93	7.3	1181	92.7	0.08	1.05	0.85	1.30	0.68	1.01	0.81	1.26	0.91	1.05	0.85	1.30	0.68
	10–12	477	7.0	6364	93.0		Reference				Reference				Reference			
	13–15	279	7.3	3517	92.7		1.05	0.91	1.22	0.47	1.03	0.89	1.19	0.70	1.05	0.91	1.22	0.47
	≥16	260	6.0	4056	94.0		**0.86**	**0.75**	**1.00**	**0.05**	0.89	0.76	1.04	0.15	**0.86**	**0.75**	**1.00**	**0.05**
**Familial characteristics**																		
**Household income (million yen)**																		
	<3.0	238	7.3	3026	92.7	0.70	1.10	0.94	1.28	0.24	1.11	0.95	1.30	0.18	1.09	0.92	1.28	0.31
	3.0–4.9	419	6.7	5879	93.3		Reference				Reference				Reference			
	5.0–7.9	249	7.0	3304	93.0		1.05	0.91	1.23	0.50	1.08	0.92	1.25	0.36	1.10	0.94	1.28	0.26
	≥8	71	6.9	959	93.1		1.04	0.81	1.32	0.78	1.11	0.87	1.43	0.40	1.14	0.88	1.47	0.32

^1^: Calculated by Chi-square test. ^2^: Calculated by generalized liner regression models. ^3^: Adjustment model was adjusted by maternal age, and maternal education. ^4^: Based on DAG model was as Figure 2 as follows: Maternal age was adjusted by maternal educational level; Maternal BMI was adjusted by maternal age, maternal active smoking, and maternal educational level; Maternal active smoking at 1st trimester was adjusted by maternal educational level, and maternal drinking habit during 1st trimester; Maternal passive smoking at 1st trimester was adjusted by paternal active smoking during 1st trimester and parental educational level; Maternal drinking habit at 1st trimester was adjusted by maternal educational level; Maternal previous medical history was adjusted by maternal age, and maternal educational level; Maternal regular use of any supplement was adjusted by maternal age, maternal previous medical history, and maternal educational level; Using ART was adjusted by maternal age, maternal educational level, and household income. Maternal educational level was not adjusted by anything. Paternal age was adjusted by paternal educational level. Paternal active smoking at 1st trimester was adjusted by maternal educational level. Paternal previous medical history was adjusted by paternal age and paternal educational level. Paternal educational level was not adjusted by anything. Household Income was adjusted by parental age and parental educational level. ^5^: Term- small for gestational age (SGA) case group was compared with a control group of infants born at 37-41 weeks’ gestational age. ART: assisted reproductive technology; BMI: body mass index; CI: Confidence Interval; DAG: directed acyclic graph; RRs: Relative Risks; term-SGA: term-Small for Gestational Age.

**Table 4:** The prevalence and the relative risk of preterm birth (*n* = 805) stratified by parental characteristics.

		PTB		Non PTB			Crude ^2^				Adjustment Model ^2,3^				Based on DAG Model ^2,4^			
								95%CI				95%CI				95%CI		
		*N*	%	*N*	%	*p*-Value ^1^	RRs	Lower	Upper	*p*-Value	RRs	Lower	Upper	*p*-Value	RRs	Lower	Upper	*p*-Value
**Maternal characteristics**																		
**Age at entry (years old)**																		
	<24	95	3.6	2539	96.4	**<0.01**	0.86	0.69	1.06	0.15	0.86	0.69	1.09	0.20	0.86	0.69	1.09	0.20
	25–34	518	4.2	11,766	95.8		Reference				Reference				Reference			
	≥35	192	6.1	2940	93.9		**1.45**	**1.24**	**1.71**	**<0.01**	**1.45**	**1.23**	**1.71**	**<0.01**	**1.45**	**1.23**	**1.71**	**<0.01**
**Prepregnancy BMI (kg/m^2^)**																		
	<18.5	1164	29.2	2827	70.8	**0.01**	**1.34**	**1.12**	**1.59**	**<0.01**	**1.39**	**1.17**	**1.65**	**<0.01**	**1.45**	**1.21**	**1.73**	**<0.01**
	18.5–24.9	505	4.1	11,795	95.9		Reference				Reference				Reference			
	25.0–29.9	64	4.8	1277	95.2		1.16	0.90	1.50	0.26	1.13	0.87	1.46	0.36	1.15	0.88	1.50	0.30
	≥30.0	17	4.7	345	95.3		1.14	0.71	1.83	0.59	1.12	0.70	1.80	0.64	0.97	0.57	1.67	0.92
**Active smoking during 1st trimester**																		
	No	642	4.5	13,783	95.5	0.75	Reference				Reference				Reference			
	Yes	91	4.6	1884	95.4		1.04	0.84	1.28	0.75	1.04	0.83	1.30	0.73	1.03	0.83	1.29	0.76
**Passive smoking during 1st trimester**																		
	No	203	4.2	4654	95.8	0.44	Reference				Reference				Reference			
	Yes	504	4.4	10,823	95.6		1.06	0.91	1.25	0.44	1.12	0.95	1.32	0.18	0.82	0.58	1.15	0.23
**Drinking habit during 1st trimester**																		
	Never	317	4.7	6371	95.3	0.27	Reference				Reference				Reference			
	Ex-drinker	358	4.2	8088	95.8		0.89	0.77	1.04	0.14	0.90	0.78	1.04	0.16	0.89	0.77	1.03	0.13
	Current drinker	83	4.1	1926	95.9		0.87	0.69	1.1	0.25	0.85	0.67	1.08	0.17	0.87	0.69	1.10	0.24
**Previous medical history**																		
	No	398	4.1	9382	95.9	**<0.01**	Reference				Reference				Reference			
	Yes	406	4.9	7854	95.1		**1.21**	**1.06**	**1.38**	**0.01**	**1.17**	**1.02**	**1.35**	**0.03**	**1.17**	**1.02**	**1.35**	**0.02**
**Regular use of any supplement**																		
	No	558	4.4	11,991	95.6	0.92	Reference				Reference				Reference			
	Yes	246	4.5	5246	95.5		1.01	0.87	1.17	0.92	1.00	0.86	1.17	0.99	0.98	0.85	1.14	0.84
**Using ART**																		
	No	703	4.2	15,862	95.8	**<0.01**	Reference				Reference				Reference			
	Yes	58	8.1	654	91.9		**1.92**	**1.48**	**2.48**	**<0.01**	**1.76**	**1.36**	**2.29**	**<0.01**	**1.56**	**1.16**	**2.09**	**<0.01**
**Maternal educational level (years)**																		
	≤9	43	4.6	891	95.4	**0.03**	1.13	0.82	1.54	0.46	1.20	0.88	1.65	0.26	1.13	0.82	1.54	0.46
	10–12	307	4.1	7205	95.9		Reference				Reference				Reference			
	13–15	329	4.7	6639	95.3		1.16	0.99	1.35	0.06	1.12	0.96	1.31	0.14	1.16	0.99	1.35	0.06
	≥16	79	4.3	1773	95.7		1.04	0.82	1.33	0.73	1.00	0.78	1.27	0.98	1.04	0.82	1.33	0.73
**Paternal characteristics**																		
**Age at entry (years old)**																		
	<24	48	3.7	1265	96.3	**0.01**	0.88	0.65	1.18	0.37	0.99	0.70	1.39	0.95	0.92	0.69	1.24	0.59
	25-34	433	4.2	9960	95.8		Reference				Reference				Reference			
	≥35	269	5.0	5071	95.0		**1.21**	**1.04**	**1.40**	**0.01**	1.05	0.88	1.25	0.58	**1.22**	**1.05**	**1.42**	**0.01**
**Smoking habit during 1st trimester**																		
	No	203	4.0	4818	96.0	0.12	Reference				Reference				Reference			
	Yes	463	4.6	9608	95.4		1.14	0.97	1.34	0.12	**1.18**	**1.00**	**1.40**	**0.05**	1.16	0.98	1.37	0.09
**Previous medical history**																		
	No	477	4.2	10,871	95.8	**0.03**	Reference				Reference				Reference			
	Yes	328	4.9	6377	95.1		**1.16**	**1.01**	**1.34**	**0.03**	1.11	0.96	1.28	0.17	1.13	0.97	1.30	0.12
**Paternal educational level (years)**																		
	≤9	51	3.8	1278	96.2	**0.04**	0.94	0.7	1.26	0.67	0.96	0.71	1.30	0.80	0.94	0.70	1.26	0.67
	10–12	292	4.1	6857	95.9		Reference				Reference				Reference			
	13–15	207	5.2	3804	94.8		**1.26**	**1.06**	**1.5**	**0.01**	**1.25**	**1.05**	**1.49**	**0.01**	**1.26**	**1.06**	**1.50**	**<0.01**
	≥16	200	4.4	4318	95.6		1.08	0.91	1.29	0.37	1.04	0.86	1.25	0.71	1.08	0.91	1.29	0.37
**Familial characteristics**																		
**Household income (million yen)**																		
	<3.0	120	3.5	3271	96.5	**0.03**	**0.80**	**0.65**	**0.98**	**0.03**	0.83	0.67	1.02	0.08	**0.80**	**0.64**	**1.00**	**0.04**
	3.0–4.9	294	4.5	6311	95.5		Reference				Reference				Reference			
	5.0–7.9	179	4.8	3557	95.2		1.08	0.9	1.29	0.43	1.03	0.86	1.24	0.75	1.02	0.84	1.23	0.84
	≥8	59	5.4	1030	94.6		1.16	0.88	1.53	0.31	1.10	0.83	1.47	0.51	1.10	0.82	1.48	0.53

^1^: Calculated by Chi-square test. ^2^: Calculated by generalized liner regression models. ^3^: Adjustment model was adjusted by maternal age, and maternal education. ^4^: Based on DAG model was as Figure 2 as follows: Maternal age was adjusted by maternal educational level; Maternal BMI was adjusted by maternal age, maternal active smoking, and maternal educational level; Maternal active smoking at 1st trimester was adjusted by maternal educational level, and maternal drinking habit during 1st trimester; Maternal passive smoking at 1st trimester was adjusted by paternal active smoking during 1st trimester and parental educational level; Maternal drinking habit at 1st trimester was adjusted by maternal educational level; Maternal previous medical history was adjusted by maternal age, and maternal educational level; Maternal regular use of any supplement was adjusted by maternal age, maternal previous medical history, and maternal educational level; Using ART was adjusted by maternal age, maternal educational level, and household income; Maternal educational level was not adjusted by anything; Paternal age was adjusted by paternal educational level; Paternal active smoking at 1st trimester was adjusted by maternal educational level; Paternal previous medical history was adjusted by paternal age and paternal educational level; Paternal educational level was not adjusted by anything; Household Income was adjusted by parental age and parental educational level. ART: assisted reproductive technology; BMI: body mass index; CI: Confidence Interval; DAG: directed acyclic graph; PTB: Preterm birth; RRs: Relative Risks.

**Table 5:** Stratified analysis by interaction covariates to examine parental risk factors for preterm birth and very low birth weight.

			Case		Non Case		Based on DAG Model ^1,2^			
								95%CI		
			*N*	%	*N*	%	RRs	Lower	Upper	*p*-Value
**VLBW**										
**Maternal age at entry (years old)**	*	**Maternal educational level (years)**								
<24	*	≤9	2	0.5	419	99.5	1.37	0.27	7.06	0.71
	*	10–12	5	0.3	1441	99.7	Reference			
	*	13–15	2	0.4	562	99.6	1.03	0.20	5.27	0.98
	*	≥16	0	0.0	73	100.0	NA	NA	NA	NA
25-34	*	≤9	2	0.5	427	99.5	1.52	0.35	6.62	0.60
	*	10–12	15	0.3	4873	99.7	Reference			
	*	13–15	14	0.3	5069	99.7	0.90	0.43	1.86	0.77
	*	≥16	8	0.6	1348	99.4	1.92	0.82	4.52	0.15
≥35	*	≤9	0	0.0	84	100.0	NA	NA	NA	NA
	*	10–12	10	0.9	1163	99.1	Reference			
	*	13–15	7	0.5	1312	99.5	0.62	0.24	1.63	0.33
	*	≥16	2	0.5	422	99.5	0.56	0.12	2.53	0.42
**PTB**										
**Maternal educational level (years)**	*	**Prepregnancy BMI (kg/m^2^)**								
≤9	*	<18.5	13	6.7	180	93.3	**2.31**	**1.15**	**4.65**	**0.02**
	*	18.5–24.9	21	3.6	556	96.4	Reference			
	*	25.0–29.9	4	4.4	86	95.6	1.31	0.45	3.79	0.63
	*	≥30.0	3	9.7	28	90.3	1.60	0.39	6.60	0.52
10–12	*	<18.5	71	5.7	1178	94.3	**1.72**	**1.31**	**2.26**	**<0.01**
	*	18.5–24.9	191	3.6	5054	96.4	Reference			
	*	25.0–29.9	35	5.4	608	94.6	1.45	1.01	2.10	0.06
	*	≥30.0	3	1.5	191	98.5	NA	NA	NA	NA
13–15	*	<18.5	59	4.9	1150	95.1	1.06	0.79	1.43	0.69
	*	18.5–24.9	236	4.7	4814	95.3	Reference			
	*	25.0–29.9	24	4.8	478	95.2	1.00	0.65	1.54	0.99
	*	≥30.0	9	7.8	106	92.2	1.64	0.84	3.24	0.19
≥16	*	<18.5	21	6.4	307	93.6	**1.87**	**1.12**	**3.12**	**0.02**
	*	18.5–24.9	55	4.0	1325	96.0	Reference			
	*	25.0–29.9	1	1.0	102	99.0	0.27	0.04	1.94	0.19
	*	≥30.0	2	10.0	18	90.0	2.78	0.73	10.59	0.13
**Maternal age at entry (years old)**	*	**Using ART**								
<24	*	no	88	3.5	2395	96.5	Reference			
	*	yes	2	9.1	20	90.9	3.05	0.80	11.62	0.18
25–34	*	no	451	4.0	10,890	96.0	Reference			
	*	yes	39	9.2	386	90.8	**2.06**	**1.45**	**2.93**	**<0.01**
≥35	*	no	164	6.0	2570	94.0	Reference			
	*	yes	17	6.4	247	93.6	0.98	0.59	1.65	0.95

^1^: Calculated by generalized liner regression models. ^2^: Based on DAG model was as Figure 2 as follows: Maternal age was adjusted by maternal educational level; Maternal educational level was not adjusted by anything. ART: assisted reproductive technology; BMI: body mass index; CI: Confidence Interval; DAG: directed acyclic graph; PTB: Preterm birth; RRs: Relative Risks; VLBW: Very Low Birth Weight.

## Supplementary Materials

The following are available online at http://www.mdpi.com/1660-4601/15/2/369/s1, Table S1: The parental characteristics of participants imputed missing values, Table S2: Parental characteristics by very low birth weight (*n* = 72), term-small for gestational age (*n* = 1192), births and preterm birth (*n* = 805), and imputed missing values, Table S3: The relative risks of very low birth weight (*n* = 72), term-small for gestational age (*n* = 1192), births and preterm birth (*n* = 805), and imputed missing values, stratified by parental characteristics.
