# Supplementary material for: Different Risk Factors for Very Low Birth Weight, Term-Small-for-Gestational-Age, or Preterm Birth in Japan
Source: Int J Environ Res Public Health. 2018 Feb 21;15(2):369. doi: 10.3390/ijerph15020369 (PMC5858438; doi:10.3390/ijerph15020369)
Supplement: Supplementary file 1 [file ijerph-15-00369-s001.pdf]

Supplementary Materials: Different Risk Factors for Very Low Birth Weight, Term-Small-for-Gestational-Age, or Preterm Birth in Japan

Naomi Tamura, Tomoyuki Hanaoka, Kumiko Ito, Atsuko Araki, Chihiro Miyashita, Sachiko Ito, Hisanori Minakami, Kazutoshi Cho, Toshiaki Endo, Kazuo Sengoku, Katsuhiko Ogasawara and Reiko Kishi *

Table S1. The parental characteristics of participants imputed missing values.

	N (%)			*N* (%)	
**Maternal characteristics**			**Paternal characteristics**		
**Age at entry (years old)**			**Age at entry (years old)**		
≤24	2635	(14.6)	≤24	1320	(7.3)
25–34	12290	(68.1)	25–34	11399	(63.1)
≥35	3134	(17.4)	≥35	5340	(29.6)
**Prepregnancy BMI (kg/m²)**			**Smoking habit during 1st trimester**		
<18.5	3054	(16.9)	No	6451	(35.7)
18.5–24.9	13297	(73.6)	Yes	1734	(9.6)
25.0-29.9	1346	(7.5)	**Previous medical history**		
≥30.0	362	(2.0)	No	11351	(62.9)
**Active smoking during 1st trimester**			Yes	6708	(37.1)
No	15846	(87.7)	**Paternal educational level (years)**		
Yes	2213	(12.3)	≤9	1332	(7.4)
**Passive smoking during 1st trimester**			10–12	7864	(43.5)
No	5603	(31.0)	13–15	4144	(22.9)
Yes	12456	(69.0)	≥16	4719	(26.1)
**Drinking habit during 1st trimester**			**Familial characteristics**		
Never	7153	(39.6)	**Household income (million yen)**		

Ex-drinker	8794	(48.7)	<3.0	4612	(25.5)
Current drinker	2112	(11.7)	3.0–4.9	8141	(45.1)
**Previous medical history**			5.0–7.9	4220	(23.4)
No	9783	(54.2)	≥8	1086	(6.0)
Yes	8276	(45.8)			
**Regular use of any supplement**					
No	12565	(69.6)			
Yes	5494	(30.4)			
**Using ART**					
No	17347	(96.1)			
Yes	712	(3.9)			
**Maternal educational level (years)**					
≤9	934	(5.2)			
10–12	7825	(43.3)			
13–15	7446	(41.2)			
≥16	1854	(10.3)			

ART, assisted reproductive technology; BMI, body mass index.

Table S2. Parental characteristics by very low birth weight (*n*=72), term-small for gestational age (*n* = 1192) births, and preterm birth (*n*=805) imputed missing values.

	VLBW					Term-SGA					PTB				
	Case		Non case			Case		Non case			Case		Non case		
	N	%	*N*	%	p-value	*N*	%	*N*	%	p-value	*N*	%	*N*	%	p-value
**Number of case and non case**	72	0.4	17987	99.6		1192	6.9	16028	93.1		805	4.5	17254	95.5	
**Maternal characteristics**															
Age at entry (years old)															
<24	9	0.3	2626	99.7		158	6.2	2376	93.8		95	3.6	2539	96.4	
25-34	42	0.3	12248	99.7	**0.03**	826	7.0	10924	93.0	0.34	518	4.2	11766	95.8	**<0.01**
≥35	21	0.7	3113	99.3		208	7.1	2728	92.9		192	6.1	2940	93.9	
Prepregnancy BMI (kg/m^2)															
<18.5	11	0.4	3043	99.6		311	10.8	2574	89.2		168	5.5	2886	94.5	
18.5-24.9	50	0.4	13247	99.6	0.15	810	6.4	11900	93.6	**<0.01**	556	4.2	12741	95.8	**0.01**
25.0-29.9	7	0.5	1339	99.5		55	4.3	1225	95.7		64	4.8	1282	95.2	
≥30.0	4	1.1	358	98.9		16	4.6	329	95.4		17	4.7	345	95.3	
Active smoking during 1st trimester															
No	60	0.4	15786	99.6	0.25	1051	7.0	14062	93.0	0.66	706	4.5	15140	95.5	0.97
Yes	12	0.5	2201	99.5		141	6.7	1966	93.3		99	4.5	2114	95.5	
Passive smoking during 1st trimester															
No	16	0.3	5587	99.7	0.11	334	6.2	5024	93.8	**0.02**	238	4.2	5365	95.8	0.36
Yes	56	0.4	12400	99.6		858	7.2	11004	92.8		567	4.6	11889	95.4	
Drinking habit during 1st trimester															
Never	21	0.3	7132	99.7		424	6.2	6377	93.8		343	4.8	6810	95.2	
Ex-drinker	41	0.5	8753	99.5	0.19	570	6.8	7832	93.2	**<0.01**	373	4.2	8421	95.8	0.20
Current drinker	10	0.5	2102	99.5		198	9.8	1819	90.2		89	4.2	2023	95.8	
Previous medical history															

No	38	0.4	9745	99.6	0.81	642	6.9	8724	93.1	0.70	398	4.1	9385	95.9	**<0.01**
Yes	34	0.4	8242	99.6		550	7.0	7304	93.0		407	4.9	7869	95.1	
Regular use of any supplement															
No	50	0.4	12515	99.6	0.98	802	6.7	11178	93.3	0.08	559	4.4	12006	95.6	0.93
Yes	22	0.4	5472	99.6		390	7.4	4850	92.6		246	4.5	5248	95.5	
Using ART															
No	65	0.4	17282	99.6	**0.01**	1142	6.9	15424	93.1	0.46	747	4.3	16600	95.7	**<0.01**
Yes	7	1.0	705	99.0		50	7.6	604	92.4		58	8.1	654	91.9	
Maternal educational level (years)															
≤9	4	0.4	930	99.6	0.70	74	8.3	813	91.7	**<0.01**	43	4.6	891	95.4	0.25
10–12	32	0.4	7793	99.6		515	6.9	6967	93.1		325	4.2	7500	95.8	
13–15	26	0.3	7420	99.7		512	7.2	6565	92.8		358	4.8	7088	95.2	
≥16	10	0.5	1844	99.5		91	5.1	1683	94.9		79	4.3	1775	95.7	
**Paternal characteristics**															
Age at entry (years old)															
<24	5	0.4	1315	99.6	**0.04**	90	7.1	1179	92.9	0.93	49	3.7	1271	96.3	**0.03**
25-34	36	0.3	11363	99.7		748	6.9	10141	93.1		287	2.6	10912	97.4	
>=35	31	0.6	5309	99.4		354	7.0	4708	93.0		269	5.0	5071	95.0	
Smoking habit during 1st trimester															
No	19	0.3	6432	99.7	0.10	396	6.4	5782	93.6	0.05	262	4.1	6189	95.9	0.05
Yes	53	0.5	11555	99.5		796	7.2	10246	92.8		543	4.7	11065	95.3	
Previous medical history															
No	46	0.4	11305	99.6	0.86	736	6.8	10116	93.2	0.34	477	4.2	10874	95.8	**0.03**
Yes	26	0.4	6682	99.6		456	7.2	5912	92.8		328	4.9	6380	95.1	

Paternal educational level (years)															
≤9	6	0.5	1326	99.5		93	7.3	1181	92.7		51	3.8	1281	96.2	
10–12	31	0.4	7833	99.6	0.99	538	7.2	6979	92.8	**0.03**	328	4.2	7536	95.8	**0.04**
13–15	16	0.4	4128	99.6		291	7.4	3628	92.6		216	5.2	3928	94.8	
≥16	19	0.4	4700	99.6		270	6.0	4237	94.0		210	4.5	4509	95.5	
**Familial characteristics**															
Household Income at entry (million yen)															
<3.0	14	0.3	4598	99.7		320	7.2	4111	92.8		168	3.6	4444	96.4	
3.0–4.9	33	0.4	8108	99.6	0.54	523	6.7	7227	93.3	0.81	375	4.6	7766	95.4	**0.01**
5.0–7.9	21	0.5	4199	99.5		278	6.9	3731	93.1		206	4.9	4014	95.1	
≥8	4	0.4	1082	99.6		71	6.9	959	93.1		56	5.2	1030	94.8	

1: Calculated by Chi-square test. 2: Term- small for gestational age (term-SGA) case group were compared with a control group of infants born at 37-41 weeks' gestational age. ART, assisted reproductive technology; BMI, body mass index; PTB, preterm birth; RRs, relative risks; term-SGA, term- small for gestational age; VLBW, very low birth weight.

Table S3. The relative risks of very low birth weight (*n* = 72), term-small for gestational age (*n* = 1192), preterm birth (*n* = 805) imputed missing values, stratified by parental characteristics [1].

	**VLBW**										**Term-SGA**										**PTB**									
			**Crude**				**Based on DAG model[2]**						**Crude**				**Based on DAG model[2]**						**Crude**				**Based on DAG model[2]**			
	**N**	**%**	**RRs**	**95%CI lower**	**95%CI upper**	**p-value**	**RRs**	**95%CI lower**	**95%CI upper**	**p-value**	**N**	**%**	**RRs**	**95%CI lower**	**95%CI upper**	**p-value**	**RRs**	**95%CI lower**	**95%CI upper**	**p-value**	**N**	**%**	**RRs**	**95%CI lower**	**95%CI upper**	**p-value**	**RRs**	**95%CI lower**	**95%CI upper**	**p-value**
**Maternal characteristics**																														
**Age at entry (years)**																														
<24	9	0.3	1.00	0.49	2.05	1.00	0.97	0.46	2.04	0.94	158	6.2	0.89	0.75	1.05	0.15	0.85	0.71	1.00	0.05	95	3.6	0.86	0.69	1.06	0.15	0.86	0.69	1.07	0.16
25–34	42	0.3	Reference				Reference				826	7.0	Reference				Reference				518	4.2	Reference				Reference			
≥35	21	0.7	**1.96**	**1.16**	**3.31**	**0.02**	**1.95**	**1.16**	**3.29**	**0.02**	208	7.1	1.01	0.87	1.17	0.92	1.02	0.88	1.18	0.81	192	6.1	**1.45**	**1.24**	**1.71**	**<0.01**	**1.46**	**1.24**	**1.71**	**<0.01**
**BMI (kg/m²)**																														
<18.5	11	0.4	0.96	0.50	1.84	0.90	1.00	0.52	1.92	0.99	311	10.8	**1.69**	**1.49**	**1.92**	**<0.01**	**1.71**	**1.51**	**1.94**	**<0.01**	168	5.5	**1.32**	**1.11**	**1.56**	**<0.01**	**1.36**	**1.15**	**1.61**	**<0.01**
18.5–25.0	50	0.4	Reference				Reference				810	6.4	Reference				Reference				556	4.2	Reference				Reference			
25.0-29.9	7	0.5	1.38	0.63	3.04	0.44	1.30	0.59	2.86	0.53	55	4.3	**0.67**	**0.52**	**0.88**	**<0.01**	**0.66**	**0.51**	**0.86**	**<0.01**	64	4.8	1.14	0.88	1.46	0.33	1.10	0.85	1.41	0.48
≥30.0	4	1.1	2.94	1.07	8.09	0.07	2.78	1.01	7.70	0.09	16	4.6	0.73	0.45	1.18	0.17	0.71	0.44	1.15	0.14	17	4.7	1.12	0.70	1.80	0.64	1.10	0.69	1.76	0.69
**Active smoking**																														
No	60	0.4	Reference				Reference				1051	7.0	Reference				Reference				706	4.5	Reference				Reference			
Yes	12	0.5	1.43	0.77	2.66	0.27	1.40	0.76	2.61	0.30	141	6.7	0.96	0.81	1.14	0.66	0.95	0.80	1.13	0.56	99	4.5	1.00	0.82	1.23	0.97	1.01	0.83	1.25	0.89
**Passive smoking**																														
No	16	0.3	Reference				Reference				334	6.2	Reference				Reference				238	4.2	Reference				Reference			
Yes	56	0.4	1.57	0.90	2.74	0.10	1.30	0.45	3.73	0.63	858	7.2	**1.16**	**1.03**	**1.31**	**0.02**	1.14	0.90	1.44	0.27	567	4.6	1.07	0.92	1.24	0.36	0.80	0.59	1.10	0.16

**Drinking habit**																														
Never	21	0.3	Reference				Reference				424	6.2	Reference				Reference				343	4.8	Reference				Reference			
Ex-drinker	41	0.5	1.59	0.94	2.68	0.08	1.57	0.93	2.66	0.09	570	6.8	1.09	0.96	1.23	0.17	1.10	0.97	1.24	0.12	373	4.2	0.88	0.77	1.02	0.09	0.88	0.77	1.02	0.10
Current drinker	10	0.5	1.61	0.76	3.42	0.23	1.59	0.75	3.37	0.24	198	9.8	**1.57**	**1.34**	**1.85**	**<0.01**	**1.58**	**1.34**	**1.85**	**<0.01**	89	4.2	0.88	0.70	1.10	0.26	0.89	0.70	1.11	0.29
**Medical history**																														
No	38	0.4	Reference				Reference				642	6.9	Reference				Reference				398	4.1	Reference				Reference			
Yes	34	0.4	1.06	0.67	1.68	0.81	1.06	0.67	1.68	0.81	550	7.0	1.02	0.92	1.14	0.70	1.02	0.91	1.14	0.71	407	4.9	**1.21**	**1.06**	**1.38**	**<0.01**	**1.19**	**1.04**	**1.36**	**0.01**
**Regular use of any supplement**																														
No	50	0.4	Reference				Reference				802	6.7	Reference				Reference				559	4.4	Reference				Reference			
Yes	22	0.4	1.01	0.61	1.66	0.98	0.95	0.57	1.58	0.85	390	7.4	1.11	0.99	1.25	0.08	**1.13**	**1.00**	**1.27**	**0.05**	246	4.5	1.01	0.87	1.17	0.92	0.97	0.84	1.13	0.71
**Using ART**																														
No	65	0.4	Reference				Reference				1142	6.9	Reference				Reference				747	4.3	Reference				Reference			
Yes	7	1	**2.62**	**1.21**	**5.70**	**0.03**	2.23	1.00	4.93	0.07	50	7.6	1.11	0.84	1.46	0.46	1.12	0.85	1.47	0.44	58	8.1	**1.89**	**1.46**	**2.44**	**<0.01**	**1.70**	**1.31**	**2.21**	**<0.01**
**Maternal education (years)**																														
≤9	4	0.4	1.05	0.37	2.95	0.93	1.05	0.37	2.95	0.93	74	8.3	1.21	0.96	1.53	0.12	1.21	0.96	1.53	0.12	43	4.6	1.11	0.81	1.51	0.52	1.11	0.81	1.51	0.52
10–12	32	0.4	Reference				Reference				515	6.9	Reference				Reference				325	4.2	Reference				Reference			
13–15	26	0.3	0.85	0.51	1.43	0.55	0.85	0.51	1.43	0.55	512	7.2	1.05	0.93	1.18	0.41	1.05	0.93	1.18	0.41	358	4.8	1.16	1.00	1.34	0.05	1.16	1.00	1.34	0.05
≥16	10	0.5	1.32	0.65	2.68	0.46	1.32	0.65	2.68	0.46	91	5.1	**0.75**	**0.60**	**0.93**	**<0.01**	**0.75**	**0.60**	**0.93**	**<0.01**	79	4.3	1.03	0.81	1.30	0.84	1.03	0.81	1.30	0.84
**Paternal characteristics**																														
**Age at entry (years)**																														
<24	5	0.4	1.20	0.47	3.05	0.71	1.16	0.45	3.00	0.76	90	7.1	1.03	0.84	1.27	0.77	1.00	0.81	1.24	0.99	49	3.7	0.87	0.65	1.16	0.33	0.89	0.67	1.20	0.44
25–34	36	0.3	Reference				Reference				748	6.9	Reference				Reference				287	2.6	Reference				Reference			
≥35	31	0.6	**1.84**	**1.14**	**2.97**	**0.01**	**1.85**	**1.14**	**3.00**	**0.01**	354	7.0	1.02	0.90	1.15	0.77	1.04	0.92	1.18	0.53	269	5.0	**1.18**	**1.02**	**1.36**	**0.03**	**1.18**	**1.02**	**1.37**	**0.03**

**Smoking habit**																														
No	19	0.3	Reference				Reference				283	6.3	Reference				Reference				262	4.1	Reference				Reference			
Yes	53	0.5	1.55	0.92	2.62	0.09	1.60	0.93	2.76	0.08	796	7.2	1.09	0.96	1.23	0.18	1.09	0.96	1.23	0.18	543	4.7	1.15	1.00	1.33	0.05	**1.19**	**1.03**	**1.39**	**0.02**
**Medical history**																														
No	46	0.4	Reference				Reference				736	6.8	Reference				Reference				477	4.2	Reference				Reference			
Yes	26	0.4	0.96	0.59	1.55	0.86	0.98	0.60	1.58	0.93	456	7.2	1.06	0.94	1.18	0.35	1.06	0.95	1.19	0.30	328	4.9	**1.16**	**1.01**	**1.33**	**0.03**	1.12	0.97	1.29	0.12
**Paternal education (years)**																														
≤9	6	0.5	1.14	0.48	2.73	0.77	1.14	0.48	2.73	0.77	93	7.3	1.02	0.82	1.26	0.87	1.02	0.82	1.26	0.87	51	3.8	0.92	0.69	1.23	0.56	0.92	0.69	1.23	0.56
10–12	31	0.4	Reference				Reference				538	7.2	Reference				Reference				328	4.2	Reference				Reference			
13–15	16	0.4	0.98	0.54	1.79	0.95	0.98	0.54	1.79	0.95	291	7.4	1.04	0.90	1.19	0.60	1.04	0.90	1.19	0.60	216	5.2	**1.25**	**1.06**	**1.48**	**<0.01**	**1.25**	**1.06**	**1.48**	**<0.01**
≥16	19	0.4	1.02	0.58	1.81	0.94	1.02	0.58	1.81	0.94	270	6.0	**0.84**	**0.73**	**0.96**	**0.01**	**0.84**	**0.73**	**0.96**	**0.01**	210	4.5	1.07	0.90	1.26	0.45	1.07	0.90	1.26	0.45
**Familial characteristics**																														
**Household income (million yen)**																														
<3.0	14	0.3	0.75	0.40	1.40	0.35	0.76	0.40	1.47	0.42	320	7.2	1.07	0.94	1.22	0.32	1.08	0.94	1.24	0.30	168	3.6	**0.79**	**0.66**	**0.95**	**<0.01**	0.84	0.70	1.01	0.07
3.0–4.9	33	0.4	Reference				Reference				523	6.7	Reference				Reference				375	4.6	Reference				Reference			
5.0–7.9	21	0.5	1.23	0.71	2.12	0.47	1.12	0.63	1.98	0.70	278	6.9	1.03	0.89	1.18	0.70	1.06	0.92	1.23	0.40	206	4.9	1.06	0.90	1.25	0.49	1.04	0.87	1.23	0.69
≥8.0	4	0.4	0.91	0.32	2.56	0.85	0.75	0.26	2.21	0.60	71	6.9	1.02	0.80	1.30	0.86	1.11	0.87	1.43	0.40	56	5.2	1.12	0.85	1.47	0.43	1.09	0.82	1.45	0.54

1: Calculated by generalized liner regression models. 2: Based on DAG model was as Figure 2 as follows: Maternal age was adjusted by maternal educational level; Maternal BMI was adjusted by maternal age, maternal active smoking, and maternal educational level; Maternal active smoking at 1st trimester was adjusted by maternal educational level, and maternal drinking habit during 1st trimester; Maternal passive smoking at 1st trimester was adjusted by paternal active smoking during 1st trimester and parental educational level; Maternal drinking habit at 1st trimester was adjusted by maternal educational level; Maternal previous medical history was adjusted by maternal age, and maternal educational level; Maternal regular use of any supplement was adjusted by maternal age, maternal previous medical history, and maternal educational level; Using ART was adjusted by maternal age, maternal educational level, and household income; Maternal educational level was not adjusted by anything; Paternal age was adjusted by paternal educational level; Paternal active smoking

at 1st trimester was adjusted by maternal educational level; Paternal previous medical history was adjusted by paternal age and paternal educational level; Paternal educational level was not adjusted by anything; Household Income was adjusted by parental age and parental educational level. ART, assisted reproductive technology; BMI, body mass index; PTB, preterm birth; RRs, relative risks; term-SGA, term- small for gestational age; VLBW, very low birth weight.

© 2018 by the authors. Submitted for possible open access publication under the terms and conditions of the Creative Commons Attribution (CC BY) license (http://creativecommons.org/licenses/by/4.0/).
